# RAREsim2: flexible simulation of rare variant genetic data using real haplotypes

**DOI:** 10.1093/bioinformatics/btag241

**Published:** 2026-05-04

**Authors:** Jessica I Murphy, Ryan Barnard, Megan Null, Audrey E Hendricks

**Affiliations:** Department of Biostatistics and Informatics, University of Colorado Anschutz, Aurora, CO 80045, United States; Department of Mathematics and Physical Sciences, The College of Idaho, Caldwell, ID 83605, United States; Department of Mathematics, Boise State University, Boise, ID 83725, United States; Department of Biostatistics and Informatics, University of Colorado Anschutz, Aurora, CO 80045, United States; Department of Biomedical Informatics, University of Colorado Anschutz, Aurora, CO 80045, United States; Department of Mathematical and Statistical Sciences, University of Colorado Denver, Denver, CO 80217, United States; Human Medical Genetics and Genomics Program, University of Colorado Anschutz, Aurora, CO 80045, United States

## Abstract

**Motivation:**

Realistic simulated data is critical for advancing methodological development and optimizing study design in genetics research. However, many genetic simulation tools are unable to replicate the distribution of rare variants or incorporate key genetic information, such as functional annotations and linkage disequilibrium. RAREsim, an accurate rare variant simulation algorithm that uses real genetic haplotypes, was developed to address these limitations. Here, we introduce RAREsim2, an update that provides both streamlined software and new functionalities for simulating individual-level differences (e.g., case-control status, technological or batch effects) and variant-level differences to represent a variety of causal models.

**Results:**

We demonstrate RAREsim2’s utility with three rare variant association methods (Burden, SKAT, and SKAT-O) across several simulation scenarios, including various genetic ancestries, gene sizes, strengths of association, and proportions of risk variants. Type I Error was maintained and the test with the highest power matched previously known patterns. Importantly, real genetic regions can be simulated to include known variant functions and disease associations. Ultimately, RAREsim2 offers additional flexibility and ease in simulating a multitude of realistic genetic scenarios.

**Availability and implementation:**

The RAREsim2 Python package is publicly available on Github (https://github.com/Hendricks-Research-Team/RAREsim2), PyPI (https://pypi.org/project/raresim/), and Zenodo (https://doi.org/10.5281/zenodo.19442523). Code for the example demonstration can be found at https://github.com/JessMurphy/RAREsim2-demo.

## 1 Introduction

Simulating data that adequately emulates reality is crucial for study design, power analyses, and method development and evaluation. However, in genetics research, simulating a realistic distribution of rare variants (defined here as variants with a minor allele frequency [MAF] < 1%) while preserving variant level information, such as functional variant annotations and disease associations, is particularly challenging. In 2022, we developed the simulation method and software RAREsim to address these gaps. RAREsim uses real genetic haplotypes to create simulated data that matches the observed AF distributions and linkage disequilibrium (LD) patterns seen in real data. Thus, RAREsim can capture unique characteristics of specific genetic regions and integrate existing variant features such as functional annotations, constrained regions, and disease loci (Null et al. 2022). Despite these advances, gaps remain including the ability to simulate case-control status or other group-level differences.

Here, we present RAREsim2, which streamlines the software and provides additional functionalities. These new capabilities include simulating different groups of individuals, such as cases and controls, to facilitate power analyses and incorporating technological or batch effects. RAREsim2 also supports defining and simulating distinct groups of variants, allowing researchers to model diverse genetic architectures (e.g., causal variants with opposite directions of effect). Below, we describe the RAREsim2 workflow, provide exemplars of its utility, and benchmark its performance. Ultimately, RAREsim2 enables the simulation of rare variant genetic data across a variety of scenarios using a simple and flexible pipeline that can be tailored to fit users’ specific needs.

## 2 Improvements and updates

### 2.1 Workflow for simulating different groups of individuals with distinct variant structures

RAREsim2 can simulate differences within groups of individuals (e.g., cases and controls, datasets from different sources) based on the general workflow below.

1. *Prepare* input files.
  - *Simulate* haplotypes at the population-level with an over-abundance of rare variants using HAPGEN2 (Su et al. 2011). Simulate all haplotypes together to avoid batch effects and inflated type I error.
  - *Annotate* the legend file with variant-level information (e.g., user-specified functional annotations, risk or protective status).
  - *Estimate* the expected number of functional ($E_{Bin_{j},\;\mathrm{fun}}[v]$) and synonymous ($E_{Bin_{j},\;\mathrm{syn}}[v]$) rare variants for each minor allele count (MAC) bin, j, using the *calc* function.
    i. Use weights $w_{\mathrm{fun}}$ and $w_{\mathrm{syn}}$ (default of 1) to scale the expected number of user-annotated functional and synonymous variants, respectively, within each group (e.g. $w_{\mathrm{fun}}=1.2$ for cases and $w_{\mathrm{fun}}=1$ for controls).
2. *Prune* the over-simulated haplotype dataset to match the expected distributions of functional ($w_{\mathrm{fun}}{*\;E}_{Bin_{j},\;\mathrm{fun}}[v]$) and synonymous ($w_{\mathrm{syn}}*\;E_{Bin_{j},\;\mathrm{syn}}[v]$) variants using the *sim* function.
3. *Extract* subsets of 2N haplotypes, where N is the simulated sample size (e.g., $2N_{\mathrm{case}}$ or ${2N}_{\mathrm{control}}$) using the *extract* function.

Steps 1a and 1b are performed prior to using the RAREsim2 *calc*, *sim*, and *extract* functions in Steps 1c, 2, and 3, respectively. Steps 2–3 can be repeated to generate additional datasets (e.g., cases, controls) or association scenarios (e.g., type I error, power) for the desired study design. While functional variants typically refer to protein-altering variants (e.g., missense and loss-of-function) and synonymous variants do not alter the amino acid sequence, RAREsim2 allows users to specify variant annotations, including functional status, providing substantial flexibility.

**Figure 1:**
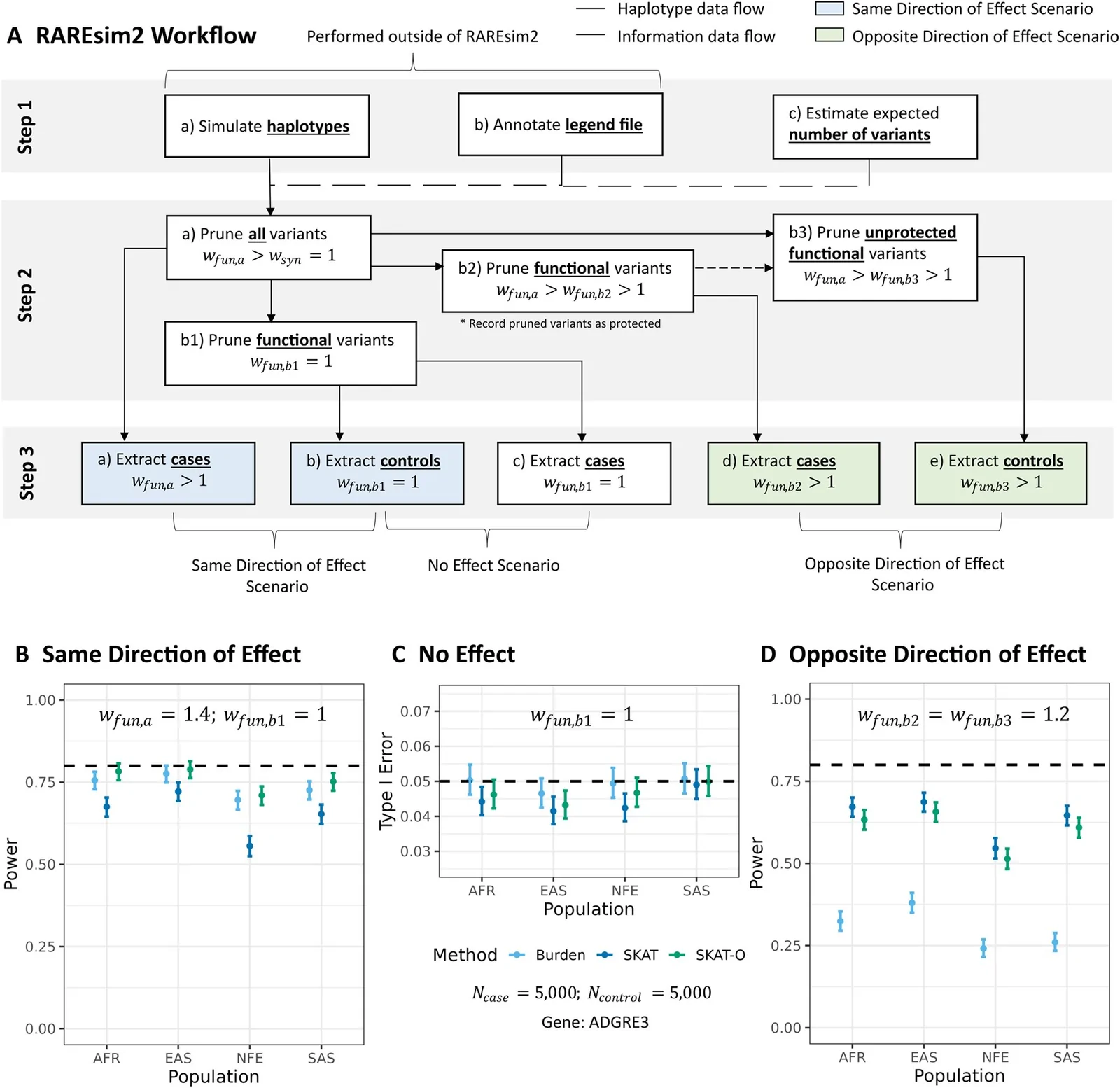
**RAREsim2’s general workflow and benchmarking. (A) General Workflow**. Flowchart for generating datasets for three simulation scenarios: same direction of effect (i.e., Power, causal risk variants only in cases), no effect (i.e., Type I error, no causal variants), and opposite direction of effect (i.e., Power, causal risk variants in cases and causal protective variants in controls). **(B–D) Benchmarking**. Cases (*N* = 5000) and controls (*N* = 5000) were simulated for each scenario using 1000 Genomes reference data and gnomAD target data from four genetic ancestry populations: African (AFR), East Asian (EAS), Non-Finnish European (NFE), and South Asian (SAS). Datasets were created according to the flowchart with weights $w_{\mathrm{fun},a}=1.4$, $w_{\mathrm{fun},b1}=1$, and $w_{\mathrm{fun},b2}=w_{\mathrm{fun},b3}=1.2$ for Step 2. Results are shown for the *ADGRE3* gene. **(B) Same direction of effect scenario** (p=1). As expected, Burden is more powerful than SKAT. **(C) No Effect Scenario**. Type I error is well-controlled (i.e., 0.05) across the Burden, SKAT, and SKAT-O rare variant association tests. **(D) Opposite direction of effect scenario** (p=0.5). As expected, SKAT is much more powerful than Burden. SKAT-O is powerful across the two power scenarios.

A flowchart of how RAREsim2 can be used to simulate case-control data is presented in Fig. 1A, with more detailed versions in the supplementary information (Figures S1–S3, available as supplementary data at *Bioinformatics* online). The flowchart shows the over-simulated haplotypes, annotated legend file, and expected number of variants are all user inputs for the pruning process (Step 2), which outputs pruned haplotypes and an updated legend file. Random subsets of the pruned haplotypes can then be extracted and designated as cases or controls (Step 3) and/or used as input for additional pruning steps. In addition to case and control groups, other groups of individuals can be simulated, such as external controls or batch effects (Figure S3, available as supplementary data at *Bioinformatics* online) (Wojcik et al. 2022).

### 2.2 Software enhancements and capabilities

RAREsim2’s simulation workflow has been streamlined, enabling users to implement the entire RAREsim2 pipeline in a single program following haplotype simulation in HAPGEN2. Step 1c, which estimates the expected number of functional and synonymous variants for each MAC bin, can now be performed directly in Python rather than R (as in RAREsim v1). The *calc* function in Step 1c can use default parameters, user-specified parameters, or parameters estimated from the allele frequency spectrum (AFS) of summary-level target data. See the package documentation and original RAREsim paper for more details on these options (Null et al. 2022). To generate different groups of individuals, users can employ the *extract* function (Step 3) to obtain random subsets of haplotypes, eliminating the need for additional command-line functions. A user-specified random seed can be used to ensure reproducibility and consistent haplotype extraction across differently pruned datasets.

RAREsim2 also introduces a range of new functionalities. Users can now simulate a variety of causal and association scenarios based on variant characteristics designated in the legend file, including functional annotations, pruning probability, or risk or protective status. For instance, pruning can be restricted to functional variants, or protective variants can be excluded from pruning to create groups with more protective causal variants. Additional user control over the number of rare variants is also available with weights to adjust the expected number of total, functional, or synonymous variants, and thresholds to ensure a minimum number of variants observed within each MAC bin.

A complete list of RAREsim2’s new functionalities can be found in Table S1, available as supplementary data at *Bioinformatics* online.

## 3 RAREsim2 framework for case-control scenarios

We used RAREsim2 to simulate three causal variant scenarios: (1) causal risk variants only present in cases (i.e., Power, same direction of effect); (2) no causal variants (i.e., Type I Error); and (3) causal risk variants in cases and causal protective variants in controls (i.e., Power, opposite direction of effect). In Fig. 1A, we show the steps for each scenario, which are described further below.

For the same direction of effect scenario, we first pruned the haplotypes to have more functional variants than expected ($w_{\mathrm{fun},a}>1$) and approximately the same number of synonymous variants as expected ($w_{\mathrm{syn}}=1;\;$Step 2a in Fig. 1A). Next, we extracted a random subset of ${2N}_{\mathrm{case}}$ haplotypes (Step 3a in Fig. 1A). Finally, we pruned the functional variants in the haplotypes to approximate their expected distribution ($w_{\mathrm{fun},b1}=1$; Step 2b1 in Fig. 1A) and extracted a different subset of ${2N}_{\mathrm{control}}$ haplotypes (Step 3b in Fig. 1A). This resulted in cases having more rare functional variants compared to controls (Figure S1, available as supplementary data at *Bioinformatics* online).

For the no effect scenario (i.e. type I error), we extracted ${2N}_{\mathrm{case}}$ haplotypes (Step 3c in Fig. 1A) using the same haplotype indices from Step 3a and paired them with the internal controls from Step 3b. Adjusting for sample size, the expected number of functional and synonymous variants will be the same for cases and controls (Figure S1, available as supplementary data at *Bioinformatics* online).

For the opposite direction of effect scenario, we pruned the functional variants from Step 2a to have more variants than expected but fewer variants than in Step 2a ($w_{\mathrm{fun},a}>w_{\mathrm{fun},b2}>1$; Step 2b2 in Fig. 1A); the pruned variants were labeled as “protected” in the legend file. From Step 2b2, we extracted ${2N}_{\mathrm{case}}$ haplotypes using the same haplotype indices from Step 3a (Step 3d in Fig. 1A). Then, after excluding the protective functional variants from the haplotypes in Step 2a, we pruned again ($w_{\mathrm{fun},a}>w_{\mathrm{fun},b3}>1$; Step 2b3 in Fig. 1A) and extracted ${2N}_{\mathrm{control}}$ haplotypes using the haplotype indices from Step 3b (Step 3e in Fig. 1A). Note, variants pruned from the controls are considered “risk” variants because they are present in the cases but not the controls, whereas variants pruned from the cases but not the controls are considered “protective” because they are in the controls but not the cases. Cases in Step 3d and controls in Step 3e will have the same expected number of functional and synonymous variants after adjusting for sample size if ${w_{\mathrm{fun},b2}=w}_{\mathrm{fun},b3}$, although the actual variants will differ (Figure S2, available as supplementary data at *Bioinformatics* online).

To ensure the same approximate number of causal and non-causal variants in both power scenarios, the functional weights for the opposite direction of effect scenario ($w_{\mathrm{fun},b2}$, $w_{\mathrm{fun},b3}$) were calculated as a function of the weights for Step 2a ($w_{\mathrm{fun},a}$) and Step 2b1 ($w_{\mathrm{fun},b1}$) (Equation 1)


(1) \begin{align*} w_{fun,b2} &= p \ast w_{fun,a} + (1 - p) \ast w_{fun,b1} \\ w_{fun,b3} &= (1 - p) \ast w_{fun,a} + p \ast w_{fun,b1} \end{align*}

given $w_{\mathrm{fun},a}>w_{\mathrm{fun},b2/b3}>w_{\mathrm{fun},b1}$ and $w_{\mathrm{fun},b1}=w_{\mathrm{syn}}$, where p is the proportion of risk variants and 1-p is the proportion of protective variants with 0<p<1. For example, if we assume 20% of the expected number of functional variants are causal ($w_{\mathrm{fun},a}=1.2{,\;w}_{\mathrm{fun},b1}=1$) and the proportions of risk and protective variants are equal (p=0.5), then the weights for the case and control sets for the opposite direction of effect scenario would be equal ($w_{\mathrm{fun},b2}=w_{\mathrm{fun},b3}=1.1$). If the proportions of risk and protective variants are unequal (p=0.75), then the weights would be unequal ($w_{\mathrm{fun},b2}=1.15$, $w_{\mathrm{fun},b3}=1.05$). For all scenarios, the total proportion of causal variants ($w_{\mathrm{fun},a})\;$remains the same (0.1+0.1=0.15+0.05=0.2). Here, we assumed $w_{\mathrm{syn}}=w_{\mathrm{fun},b1}=1$, but any values can be used as long as the constraints are satisfied.

## 4 Benchmarking demonstration

### 4.1 Computing environment

We used a high-performance computing cluster for the simulations and ran the RAREsim2 workflow using a singularity container with HAPGEN2 version 2.2.0, Python version 3.10.6, and R version 4.2.1. See the RAREsim2-demo Github repository for more details on the nodes and cores of the computing cluster, specific versions of the R packages, and runtimes for each step of the pipeline.

### 4.2 Input data and simulation parameters

We used reference data from 1000 Genomes phase 3 (hg19) to simulate a centimorgan block on chromosome 19 with the median number of base pairs (19,029) in four ancestral populations: African (AFR), East Asian (EAS), Non-Finnish European (NFE), and South Asian (SAS) (1000 Genomes project consortium 2015). As described in the original RAREsim paper, we modified the reference haplotype and legend files to include information (e.g., genomic position, reference allele, alternate allele) at each sequencing base within the canonical coding region (Null et al. 2022). Using this modified reference data, we simulated haplotypes in HAPGEN2 with an over-abundance of rare variants (Step 1a in Fig. 1A). We annotated the simulated data using the refGene database in ANNOVAR, classifying functional variants as nonsynomous, stopgain, or stoploss variants and synonymous variants as synonymous variants. (Step 1b in Fig. 1A) (Wang et al. 2010). We then used target summary data from gnomAD v2.1 to calculate the expected number of variants for the simulated sample size (Step 1c in Fig. 1A) (Karczewski et al. 2020).

We used the weights provided in Table S2, available as supplementary data at *Bioinformatics* online, to simulate different scenarios (Step 2 in Fig. 1A) and extracted sample sizes of ${N_{\mathrm{case}}=N}_{\mathrm{control}}=5,000$ for each scenario (Step 3 in Fig. 1A). We filtered the pruned datasets to exonic functional variants with MAF < 1% in the entire sample. The number of rare functional variants in the target dataset per gene per population are provided in Table S3, available as supplementary data at *Bioinformatics* online. We assessed the concordance between the simulated and target AFS distributions in each ancestry for three genes by calculating the difference in proportions of functional variants in each MAC bin. Seven MAC bins were used: MAC = 1 (singletons), MAC = 2 (doubletons), MAC = 3–5, MAC = 6–10, MAC = 11–20, MAC = 21 to MAF = 0.5% (MAC = 100), and MAF = 0.5% (MAC = 101) to MAF = 1% (MAC = 200).

### 4.3 Rare variant association methods

We then applied several rare variant association methods to assess RAREsim2’s ability to generate realistic genetic data across a range of causal variant patterns. Two main classes of rare variant tests are burden and variance component tests (Li and Leal 2008, Wu et al. 2011). Prior work has shown that burden tests are most powerful when there is a large proportion of causal variants with the same direction of effect, while variance component tests, such as SKAT, are most powerful when there are causal variants with opposite directions of effect. Optimal tests, which combine both burden and variance component tests, such as SKAT-O, are powerful across most scenarios (Lee et al. 2012). In practice, the performance of these methods depends on variant filtering; the inclusion of non-causal variants can dilute signal and reduce power.

Here, we benchmarked RAREsim2’s performance by assessing the power and type I error of Burden, SKAT, and SKAT-O when causal variants have the same, opposite, or no effects (Lee et al. 2023). We calculated type I error and power for 10,000 and 1,000 simulation replicates per scenario, respectively.

### 4.4 Results

Simulations matched the target AFS distributions, although singletons were slightly underrepresented for the small gene (Figures S4–S6, available as supplementary data at *Bioinformatics* online). Type I error of 0.05 was well-controlled across all methods (Fig. 1C) and for genes of varying sizes (Figure S7, available as supplementary data at *Bioinformatics* online). As expected, Burden was more powerful than SKAT for the same direction of effect scenarios (Fig. 1B), whereas SKAT was substantially more powerful for the opposite direction of effect scenarios (Fig. 1D). SKAT-O maintained high power for all scenarios. These results were consistent for genes of varying sizes, with the power of each test increasing as the number of functional rare variants in a gene increased (Figure S9, available as supplementary data at *Bioinformatics* online). Overall, Burden required larger proportions of causal variants ($w_{\mathrm{fun},a}>1.2$) before it outperformed SKAT in the same direction of effect scenario. This indicates that improved performance of SKAT relative to Burden does not necessarily imply the presence of protective variants (e.g., same direction of effect scenario in Figure S8, available as supplementary data at *Bioinformatics* online), but could instead result from the presence of non-causal variants. SKAT may be more robust to the inclusion of non-causal variants given its flexibility to model different directions of effect. In comparison, SKAT remained substantially more powerful than Burden across all proportions of causal variants for the opposite direction of effect scenarios. The power of all methods decreased as the proportion of risk variants (p) decreased, with the largest decrease observed for Burden. Power increased for all methods as the number of causal variants ($w_{\mathrm{fun},a}$) increased (Figure S8–S10, available as supplementary data at *Bioinformatics* online). All trends matched expectations, supporting RAREsim2 simulations were performing as expected.

## 5 Conclusion

We present RAREsim2, a streamlined and highly flexible tool for simulating rare genetic variants from real data. The software allows users to simulate a variety of study designs (e.g., case-control, batch effects) and genetic architectures (e.g., different proportions of causal variants, directions of effect, known genetic associations, functional variant annotations). We benchmarked RAREsim2’s performance using Burden, SKAT, and SKAT-O, demonstrating its capacity to generate realistic datasets. By simulating rare variant data while preserving key genetic features, RAREsim2 enables more accurate modeling of true genomic complexity, thus supporting improved study design, method development, and evaluation.

## Supplementary Material

btag241_Supplementary_Data

## Data Availability

Example data to run RAREsim2 is provided in the Github repository (https://github.com/Hendricks-Research-Team/RAREsim2). Code to generate data for the example demonstration can be found at https://github.com/JessMurphy/RAREsim2-demo.

## Supplementary information

Supplementary figures and tables are available at *Bioinformatics* online.
