# Correction to: RAREsim2: flexible simulation of rare variant genetic data using real haplotypes

**DOI:** 10.1093/bioinformatics/btag537

**Published:** 2026-08-14

This is a correction to: Jessica I Murphy, Ryan Barnard, Megan Null, Audrey E Hendricks, RAREsim2: flexible simulation of rare variant genetic data using real haplotypes, *Bioinformatics*, Volume 42, Issue 5, May 2026, btag241, https://doi.org/10.1093/bioinformatics/btag241

The following changes have been made to the originally published article.

The list prefixes under Step 1 in **Section 2.1** have been added to read “a”, “b” and “c”, and additionally “i Use weights[…]” under 1c.

The equation in the last paragraph of **3 RAREsim2 framework for case-control scenarios** should read:


(1) \begin{align*} w_{fun,b2} &= p \ast w_{fun,a} + (1 - p) \ast w_{fun,b1} \\ w_{fun,b3} &= (1 - p) \ast w_{fun,a} + p \ast w_{fun,b1} \end{align*}

instead of:


$$w_{\mathrm{fun},b2}=p{\;*\;w}_{\mathrm{fun},a}+(1-p)*w_{\mathrm{fun},b1}$$

